# A multidimensional analysis of prognostic factors in atopic dermatitis

**DOI:** 10.3389/fmed.2025.1554669

**Published:** 2025-04-17

**Authors:** Zhenggang Liu, Mengnan Guo, Yumei Li, Hui Xu

**Affiliations:** Department of Dermatology, Affiliated Hospital of Jiangsu University, Zhenjiang, China

**Keywords:** atopic dermatitis, signal transducer and activator of transcription, general factors, IL-9-IL-18 axis, prognostic biomarkers, ceramide profiles, NKG2D

## Abstract

Atopic dermatitis (AD) is a chronic inflammatory skin condition with a high prevalence worldwide and multifaceted pathogenesis. In general, patients with moderate to severe AD often experience relapse after discontinuing treatment. Therefore, to understand the possible factors of chronic relapse of AD and to look for biological markers that predict the relapse or poor prognosis of AD will be helpful for clinical treatment. Mutations in genes such as FLG, SPINK5, STAT, KIF3A, claudin-1, Ovol1, and HLA-DRB1 offer new insights into the genetic basis of AD. Routine factors may help improve patient lifestyle, highlight the importance of environmental influences (including psychological stress), and support clinicians in optimizing anti-infective treatment strategies. The inflammatory axis (CD30–CD30L axis, IL-9-IL-18 axis) provides new insights into the inflammatory pathways of AD and may be a target for future therapies. Low NKG2D expression may have adverse effects on prognosis. Prognostic biomarkers can play an important role in treatment monitoring, disease progression and recurrence, and provide the possibility for more personalized treatment.

## 1 Introduction

Atopic dermatitis (AD) is a chronic, recurrent inflammatory skin disease that affects the quality of life of patients, characterized by recurrent eczematous skin lesions, severe pruritus and sensitive, dry skin (1). AD is a heterogenous disease with primary T helper 2 cells (Th2)/Th22 skewing, and variable Th1/Th17 contribution (2). AD is diagnosed according to the criteria developed by Hanifin& Rajka. The pathogenesis of AD is complex, and it is evident that a strong genetic predisposition (filaggrin mutations), epidermal dysfunction, skin microbiome abnormalities (*Staphylococcus aureus* colonization), immune dysregulation, and the neuroimmune system are critical in AD development (3). AD management is multifaceted, involving emollient moisturizers, topical corticosteroid, topical calcineurin inhibitor, phosphodiesterase type 4 inhibitors, antimicrobial treatment, antihistamines, phototherapy, systemic corticosteroid, systemic immunomodulatory treatments and targeted therapies (4). Nonetheless, there is no cure for AD currently, the goals of AD treatment are to reduce pruritus and skin inflammation, restore barrier function, prevent infection and establish long-term disease control. After treatment discontinuation, AD recurs in up to 70% of cases, with a 7-year recurrence rate reaching 75.9% (5). Patients with recurrence (eczema area and severity index, EASI ≥ 16) within 3 months after cessation of treatment should be considered as a recurrent AD (6). Therefore, understanding the possible factors of AD exacerbation, chronic recurrence, and looking for biomarkers to predict AD recurrence or poor treatment response will help clinical decision-making, decrease the risk of developing comorbidities related to disease progression, and reduce the costs of AD for the health system.

## 2 Gene mutations

Research has elucidated the significant role of the filaggrin gene (FLG) as a prominent genetic risk factor in atopic dermatitis (7). Loss-of-function mutations in the gene encoding the epidermal protein filaggrin can lead to the loss or reduction of filaggrin synthesis (8). The deficiency of filaggrin in AD patients impacts on the function of the epidermis and increases the risk for microbial infection or development of other atopic diseases (9). FLG mutations has been associated with more persistent disease (10). Filaggrin-2 variation is associated with more persistent atopic dermatitis in African American subjects (11).

Mutations in SPINK5 have already been shown to be responsible for the development of Netherton syndrome, which can severely affect the skin in the form of refractory severe AD. It was clearly demonstrated that rs2303067 in SPINK5 is predisposing to early onset AD, and similar to FLG mutations, rs2303067 is also predisposing to prolonged AD duration, the need for hospitalization and more affected body parts (12, 13).

Signal transducer and activator of transcription (STAT) proteins are critical mediators of cytokine signals that regulate gene expression programs underlying key processes involved in the immune system. Case analyses indicate that gain-of-function mutations in STAT5B and STAT6 may contribute to treatment-resistant severe AD (14, 15).

Genetic variation in the cilia structural gene kinesin family number 3A (KIF3A) has been associated with AD, asthma, and the atopic march by numerous studies. A study in mice found KIF3A deficiency causes skin barrier dysfunction and contributes to AD susceptibility. KIF3A is required for skin barrier homeostasis whereby decreased KIF3A skin expression causes disrupted skin barrier function and promotes development of AD (16).

Tight junctions (TJs) are cell–cell connections located under the stratum corneum (SC), which contribute to epidermal barrier integrity. Epithelial TJs are composed of proteins including claudin, occludin and zonula occludens. Claudin 1 is integral to TJs functionality in the skin barrier. Claudin 1 mutations are associated with skin xerosis, pruritus and AD development (17, 18).

Ovol1 has also been identified as a susceptibility gene for AD by genome-wide association studies, in addition to FLG (19). Ovol1 impairment downregulates FLG, contributing to AD pathogenesis (20). Ovol1 deficient in keratinocytes impairs the barrier-promoting function of aryl hydrocarbon receptor (AhR), exacerbating AD-like inflammation (21). Ovol1 deficient affect the AhR-Ovol1-Id1 regulatory axis which promotes both epidermal and immune homeostasis in the context of skin inflammation.

Human leukocyte antigen (HLA)-DRB1 was regarded as a susceptibility factor associated with AD (22). Amino acid variations at peptide-binding pockets of HLA-DRB1 were associated with the persistence of AD in African-American children (23).

## 3 General factors

AD comprises multiple endotypes with distinct inflammatory pathways influenced by disease stage, age, and ethnicity. Th2-dominant immunity is central to AD, while Th1, Th17, and Th22 pathways also contribute (24). With regard to disease stage, Th2 activity predominates during the acute phase, whereas Th1, Th17, and Th22 responses become more prominent in chronic disease (25). With aging, Th2 activity significantly declines and the expression of Th1 and Th17 related markers increases. Elderly AD patients show reduced Th2/Th22 and increased Th1/Th17 inflammation in both the skin and blood, still presents as a type 2 inflammatory dominant disease (26). A single-center prospective cohort finding that age > 60 predicted poor response of dupilumab (27). It is speculated that the endotype shift in the elderly may be one of the reasons for the poor outcome. In the meantime, this cohort is a cross-sectional study, although there are data suggesting that type 2 inflammation decreases with age, there are no data in clinical practice suggesting that the efficacy of these drugs decreases over time. Ethnic differences further shape AD endotypes. Asian AD patients exhibit mixed Th2/Th22/Th17 inflammation, whereas white-skinned individuals predominantly display Th2/Th22-driven inflammation (26, 28). Current inclusion criteria for AD clinical trials are mostly based on disease severity rather than AD phenotyping. An attempt to define the patient’s endotype before treatment should be made to optimize therapeutic responses moving toward precision medicine based on the different clinical and molecular disease subsets.

**Figure 1:**
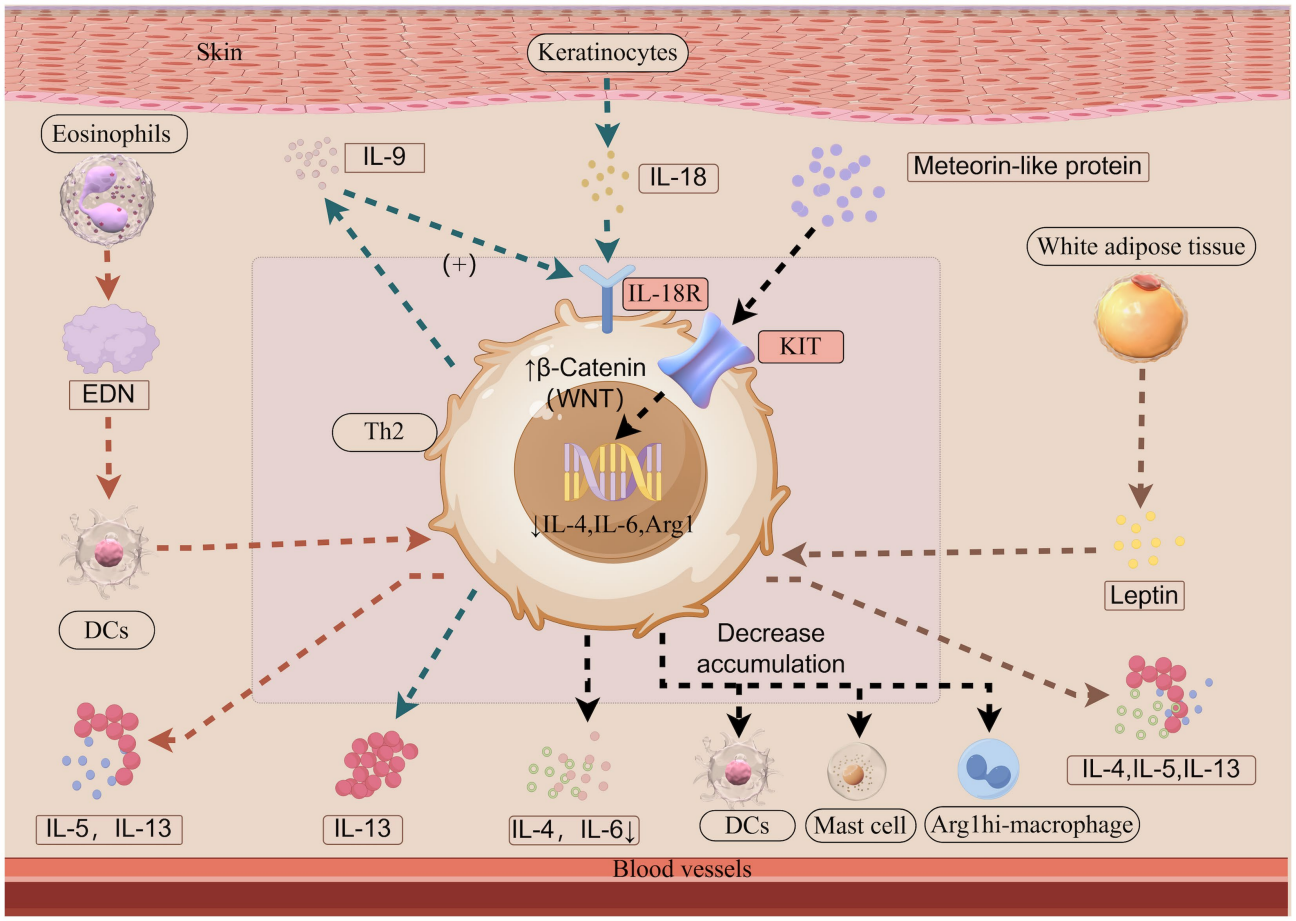
The roles of leptin, IL-9-IL-18 axis, EDN and Meteorin-like protein in atopic dermatitis (Illustration created with Figdraw). Leptin, primarily secreted by white adipose tissue, enhances Th2 responses by stimulating the release of IL-4, IL-5, and IL-13 from Th2 cells. IL-18 is predominantly produced by keratinocytes, while IL-18R is primarily expressed on T cells. The interaction between IL-18 and IL-18R activates downstream signaling pathways. IL-9 is mainly secreted by recently activated Th2 cells during the early phases of allergic skin inflammation. IL-9 promotes IL-18R expression in Th2 cells, while IL-18 functions as an upstream regulator of Th2 cells by inducing IL-13 secretion. EDN, released from eosinophil granules, activates dendritic cells, thereby amplifying antigen-specific Th2 immune responses through the enhanced secretion of IL-5 and IL-13. METRNL binds to the KIT receptor and enhances the levels of the active WNT pathway molecule β-Catenin, thereby downregulating the expression of inflammatory genes, including IL-4, IL-6, and Arginase-1 mRNA expression. Consequently, the accumulation of Arg1hi macrophages, DCs, and activated mast cells and the level of IL-4 and IL-6 are reduced. Arg1, Arginase-1; DCs, dendritic cells; EDN, eosinophil-derived neurotoxin; IL, interleukin; IL-18R, interleukin-18 receptor; Th, T helper cell type.

Obesity is considered a risk factor for AD, contributing to skin’s epidermal barrier, increased transepidermal water loss and dry skin, immune dysregulation, and alterations in the skin microbiome and gut flora, exacerbating inflammation (29). Leptin, a cytokine cascade activator secreted by white adipose tissue (30), can enhance Th2 responses, promoting interleukin (IL)-4, IL-5, and IL-13 release, immunoglobin E (IgE) production, and skin inflammation in AD (31) (Figure 1). Obesity also affects dupilumab efficacy. Higher body mass index reduces both short-term and long-term treatment effectiveness (32, 33). Real-world studies indicated that higher body mass index was associated with a lower probability of reaching an improvement of Investigator’s Global Assessment ≥2 points at Week 52 (34) and body mass index <24 was identified as potential predictive factors of good response (27). Given these associations, nutritional interventions may not only mitigate obesity-related metabolic comorbidities but also improve AD outcomes, making timely weight management essential for obese AD patients.

Head and neck dermatitis (HND) is a refractory phenotype of AD (35), that is often resistant to first-line topical therapies. And biologic treatments, such as Dupilumab, may cause the development or exacerbation of HND, defined as dupilumab-associated HND (36, 37). Malassezia overgrowth, driven by ceramide deficiency, is prevalent in HND, contributing to disease severity and treatment resistance (35, 38). In addition, the constant exposure of the head-and-neck area to several types of allergens might be a contributing factor to AD persistence (39). Persistent HND has been associated with lower dupilumab response rates (39, 40), and baseline Malassezia-specific IgE is proposed as a potential biomarker for predicting the development or exacerbation of HND (37, 41).

A positive family history of AD, particularly parental AD or other allergic diseases (e.g., rhinitis), increases the probability of the onset, recurrence and persistence of AD after puberty (42). High socioeconomic status (≥12 years of parental education), a positive skin prick test at baseline, and employment in high-risk occupations (nursing, healthcare, bakery, cleaning) are significant predictors of AD recurrence and persistence (42). Pregnancy may exacerbate AD (43). Increasing levels of progesterone and estrogen have been associated with an enhanced Th2 immune response, which supports pregnancy maintenance but may also promote atopic disease development (43, 44).

Pruritus is a common and distressing symptom of AD, with nocturnal pruritus significantly impairing sleep, leading to anxiety and daytime somnolence (45). AD skin lesions release various substances (pruritogens), including cytokines and chemokines (e.g., IL-31), and chemical mediators, triggering itching and subsequent scratching behavior. Scratching further aggravates AD, perpetuating the itch–scratch cycle. Irritation from scratching is extremely important as an exacerbation factor of AD. In addition to the therapeutic ladder for pruritus in atopic dermatitis to break the itch–scratch cycle, cutting nails short, and wearing gloves, long sleeves and long pants while sleeping, if necessary, so that scratching does not cause skin damage, may be helpful in some cases (45).

Exposure to environmental factors including allergens and stimuli in the work place and daily environment, is associated with maintenance and exacerbation of AD. Non-specific irritation present in daily-life such as contact with saliva, sweat, hair, and friction against clothes may exacerbate AD. Exposure to chlorine in water, formaldehyde, benzene, particulate matter 10 (PM10), nitrogen oxide compounds, and carbo monoxide in air was associated with AD worsening. Certain foods such as milk, eggs and wheat can trigger AD. Soap usage can thin the SC in both normal and non-lesional atopic skin, while alterations in skin pH further compromise barrier function. Additionally, disturbances in perspiration as well as excess sweat remaining on the skin surface exposed to high-temperatures and humidity may worsen symptoms of AD (46, 47).

Patients with AD have an increased risk of bacterial, viral and fungal infections of the skin, mainly due to impaired barrier function, altered immune response, certain systemic treatments and frequent scratching, which can have negative consequences on the disease course (48). Bacterial infections, particularly those caused by *Staphylococcus aureus*, are the most common and can be challenging to diagnose due to symptom overlap with AD (48). Recurrent viral infections, though less frequent, include herpes simplex virus infection, which can lead to eczema herpeticum (48). Fungal infections are also common in patients with AD. Findings suggest that Malassezia decreases the effectiveness of treatment with ruxolitinib and dupilumab (49), and *C. albicans* colonization of the gastrointestinal tract results in antigen sensitization which may potentially lead to chronic AD (48). These can also lead to infections of other organ systems including the lungs and the urinary tract. Recurrent bacterial infections can favor disease chronicisation and cause disease flares with significant morbidity, and possible mortality in patients with AD. For the management of infections in patients with AD, it is important to identify and treat the underlying infection, optimize AD treatment to improve skin barrier function and immune system regulation and implement infection prevention strategies. The risk of infection should also be considered when selecting topical or systemic immunosuppressive therapies, as some of these agents can increase the risk of viral infections.

Psychological stress contributes to skin barrier dysfunction in AD and is a well-established trigger for disease exacerbation (47). Poorly controlled AD can lead to psychological burden and secondary cognitive disturbances, reflecting the complex psycho-neuro-immunological interactions involved in eczema. Clinicians should adopt a comprehensive treatment approach that considers psychosomatic interactions. Stress-reduction interventions improve both skin manifestations and overall patient well-being (50).

Poor treatment adherence is a significant barrier to optimal outcomes in AD. Key factors contributing to nonadherence include frustration with treatment efficacy, inconvenience, and concerns about side effects. Additional barriers include forgetfulness, financial constraints, distrust in physicians, dislike of prescribed medications, and insufficient understanding of the disease or treatment (51).

Topical corticosteroids (TCS) are a common treatment for AD; however, prolonged use may increase recurrence risk. Studies have suggested that barrier defects are not repaired by steroid use and treated skin become up to 70% thinner. Additionally, TCS may increase expression of desquamatory protease KLK7, which is linked to increased AD lesions (47). TCS therapy is also associated with steroid phobia, leading to nonadherence and undertreatment. Misinformation, fear of side effects, and inadequate education contribute to this phenomenon (52). Topical steroid withdrawal syndrome (TSW), characterized by TCS dependence and skin disease worsening after TCS discontinuation, can further exacerbate steroid phobia. However, overdiagnosis of TSW may result in unnecessary treatment avoidance, leading to prolonged and intensified AD flares (53).

Patient education plays a crucial role in the long-term management of AD. Educational strategies vary, including printed materials, structured courses, and digital interventions, each influencing patient outcomes differently. Despite the prevalence of misinformation on social media, web-based interventions, smartphone applications, and teledermatology have been shown to enhance patient awareness. Additionally, social media can support online interventions, which, in some cases, may be as effective as face-to-face consultations (54).

## 4 Axis

OX40 is a co-stimulatory immune checkpoint molecule that promotes the activation and the effector function of T lymphocytes through interaction with its ligand (OX40L) on antigen-presenting cells (55). OX40–OX40L axis plays a crucial role in Th1 and Th2 cell expansion, particularly during the late phases or long-lasting response (55). Activation of the OX40–OX40L axis can promote Th2 differentiation in the absence of Th1 cytokines. A close relationship exists between this co-stimulatory pathway and the principal proinflammatory cytokines that initiate the AD inflammatory cascade, such as Thymic stromal lymphopoietin and IL-33. OX40–OX40L signaling contributes to AD chronicity by enhancing T cell expansion and survival with subsequent memory T cell development, responsible for chronic relapsing inflammation (55). OX40-OX40L pathway inhibitors, such as rocatinlimab, amlitelimab and telazorlimab, present an innovative approach by modulating T cell activity.

IL-18, produced by keratinocytes, binds interleukin-18 receptor (IL-18R) on skin immune cells, activating signaling pathways, modulating Th1/Th2 responses (56). Studies in murine models indicate that IL-18R is specifically expressed in the skin, where it triggers IL-13 secretion upon IL-18 stimulation (57). IL-9 belongs to the family of common γ chain cytokines and is linked to type 2 immunity and immunopathology (58). In human skin, IL-9 is mainly secreted by recently activated Th2 cells during early phases of allergic skin inflammation (59). IL-9 induces IL-18R expression in Th2 cells through the activation of the JAK1/JAK3-STAT1 signaling cascade. In this setting, IL-13, a well-established pathogenic cytokine in AD, production appears to be enhanced (60) (Figure 1). In conclusion, the novel IL-9-IL-18 axis provides new insights into the inflammatory pathways of AD and may represent a potential therapeutic target for its treatment. A study in mice found IL-18 blocking agent substantially controlled skin inflammation in a model of atopic dermatitis (61). However anti-IL-9 monoclonal antibody was not associated with any improvement in asthma (62).

CD30 was originally described as a Th2 cell surface-expressed molecule that also aided Th2 cell development (63). The interaction between CD30 and CD30L has been shown to regulate the generation of CD4 effector and memory T cells, in conjunction with OX40/OX40L (64, 65). CD30, similar to OX40, can activate signaling pathways, including NF-κB and PI3K/Akt, which influence T cell proliferation, survival, and cytokine production. Study confirmed upregulated expression of CD30 transcripts in CD4 T cells in the skin lesions from AD patients and found that these partially coincided with expression of CD30L, as well as with OX40 and OX40L transcripts (63). CD30–CD30L signaling may contribute to AD chronicity as well as OX40–OX40L axis.

## 5 Prognostic biomarkers

Prognostic biomarkers are used to determine the possibility or risk of progression or recurrence of a disease and the response to a given therapy. The expression of accurate and reliable biomarkers is essential for effective predictive, preventive, and personalized AD management.

AD is characterized by elevated production of the type 2 cytokines IL-4, IL-5, and IL-13, which promote AD pathogenesis. Despite initial interest in IL-5 blockade for AD treatment, clinical trials with mepolizumab (anti-IL-5 monoclonal antibody) have failed to demonstrate efficacy (66), suggesting a limited role of IL-5 in AD. In contrast, IL-31 has emerged as a key mediator of pruritus (67), while IL-4 and IL-13 remain central therapeutic targets. Dupilumab, the first biologic agent targeting IL-4 receptor α, and tralokinumab, an IL-13 inhibitor, have demonstrated significant clinical efficacy, highlighting the critical roles of IL-4 and IL-13 in AD pathogenesis and treatment. Various biological agents targeting Interleukin-4, IL-13 receptors are also being studied (68).

Total serum IgE, extensively studied as a biomarker in AD, may represent allergic diathesis rather than short-term disease activity in AD (46). High baseline IgE (≥10,000 IU/mL) was the most important patient-related factor for a poor long-term outcome, negatively impacting treatment response (69). High serum IgE levels in adult patients with AD have been associated with ongoing eczema after 10 years (70). IgE autoantibodies may serve as potential predictive biomarkers for the course of AD (71). One early study found IgE receptor antagonists (omalizumab) have not demonstrated efficacy in atopic dermatitis (72), indicating that the development of AD-like skin inflammation was largely dependent on IL-4/13 signaling but not on IgE (73). However, a small phase 2 study with 12 patients about IgE receptor antagonists showed promising results in decreasing IgE levels and percent change in Scoring Atopic Dermatitis (68). More adequate clinical studies may be needed to demonstrate a correlation between IgE receptor antagonists and AD.

Thymus and activation-regulated chemokine/chemokine C-C motif ligand 17 (TARC/CCL17) is a reliable clinical biomarker for AD severity used to monitor the efficacy of treatment (24). Elevated serum CCL17/TARC levels are associated with frequent AD relapses, even after clinical resolution (74). Persistently high TARC levels indicate inadequate treatment (75), and their increase often precedes relapse following ciclosporin tapering (76). A decrease in TARC levels to ≤837 pg/mL within 1–3 months is predictive of long-term symptom control (77). Additionally, elevated TARC levels in 2-month-old infants have been linked to a higher risk of developing AD within the initial 2 years of life (78).

Pulmonary and activation-regulated chemokine (PARC/CCL18) have been described correlating with AD severity and monitoring of disease activity during treatment (79). The rapid decline in CCL18/PARC levels was associated with good responses to abroxitinib treatment, including head and neck dermatitis (80).

Thymic stromal lymphopoietin protein is a crucial mediator of atopic inflammation, as its overexpression in murine skin models induces eczematous lesions resembling AD (81). It can induce the proliferation of Th2 memory cells that are crucial in maintaining chronic-relapse inflammation (82, 83).

Recent clinical data indicate that AD patients with low baseline lactate dehydrogenase (LDH) levels (<400 U/L) achieve higher EASI-75 response rates to dupilumab than those with elevated LDH (≥400 U/L) (84). Higher baseline LDH is also associated with poorer long-term treatment efficacy, suggesting its potential as a predictive marker for dupilumab response (33).

CXCL9 (Th1/interferon-related cytokine) and CXCL2 (Th17-related cytokine) have been suggested as treatment-specific predictive biomarkers for cyclosporine and dupilumab, respectively (85).

Macrophage-derived chemokine (MDC/CCL22) serves as the best biomarker of disease response across studies using different therapeutics (85).

The recent study found that high levels of vascular endothelial growth factor (VEGF) were associated with a higher rate of disease remission, while low VEGF has a significant impact on prediction of disease persistence (86).

Fatty-acid-binding protein 5, elevated serum eosinophil percentages and IL-13, and increased transepidermal water loss levels were identified as a potential biomarker in atopic march (87–90).

Eosinophilic granulocytes and eosinophil-derived neurotoxin (EDN) which is released from eosinophil (91) are actively involved in the regulation of allergic inflammation (92, 93). Research on dupilumab therapy revealed a significant inverse correlation between the percentage decrease in EASI scores and baseline blood eosinophil count. Furthermore, patients with low (<500/μL) baseline blood eosinophil counts showed a higher EASI-75 response rate (84). EDN can activate dendritic cells (DCs) to enhance antigen-specific Th2 immune responses, such as the release of IL-5 and IL-13 (93) (Figure 1). Research findings indicate that EDN positively correlate with the scoring AD and are notably elevated among individuals experiencing relapse. A cutoff value of 64.5 ng/mL for EDN has been identified as predictive of relapse (94).

IL-31 has been shown to induce late-onset itch in human AD patients and is thought to be involved in promoting the pathophysiology of AD and pruritus via the “scratch-itch cycle” (68). Low levels of IL-31 might predict poor efficacy of dupilumab treatment (24).

In a study based on Chinese patients, low levels of IL-36β might predict poor efficacy of dupilumab treatment (24). But, a study based on European populations has found that IL-36β levels in skin lesions of patients with AD are not increased, which may be related to cytokine profiles in the population. Even studies based on Asian populations have had conflicting results on baseline serum levels of IL-36β in patients with AD (24, 95). So the generalizability of IL-36β is still open to question.

Indoleamine 2,3-dioxygenase expression and activity in langerhans cells seems involved in the pathophysiology of eczema herpeticum in AD and could represent a predictive biomarker for patients with risk to develop eczema herpeticum and other viral complications (96).

High serum periostin and dipeptidyl peptidase-4 levels in AD patients have been reported as significant biomarkers to predict a good response to anti-IL-13 (tralokinumab) treatment (97).

Meteorin-like protein (METRNL) which is a cytokine known for its anti-inflammatory properties is significantly upregulated in the lesional skin and serum of both AD mice and patients (98). Murine studies suggest that the activation of WNT/β-catenin signaling can initiate the differentiation and expansion of CD103 + DCs (99), hamper DCs recruitment (100), decrease mast cells and pro-inflammatory factor levels (101), and drive keratinocyte proliferation for tissue repair (102). Additionally, study in murine models indicates that METRNL can bind to KIT receptor, a receptor tyrosine kinase type III (103), to mitigate allergic inflammation in AD. This effect is achieved by inhibiting immune cell expansion, enhancing β-catenin activation, and downregulating Th2-related gene expression, including IL-4, IL-6, and Arginase-1 mRNA expression. Consequently, the accumulation of Arg1^hi^ macrophages, DCs, and activated mast cells, as well as the levels of IL-4 and IL-6, are reduced (98) (Figure 1). METRNL appears to positively affect patient outcomes, its precise mechanisms require further investigation.

Ceramides in the SC play critical roles in cutaneous barrier function. A recent study found that the carbon chain lengths of ceramides containing nonhydroxy fatty acids and dihydrosphingosines (NDS), ceramides containing nonhydroxy fatty acids and sphingosines (NS), and ceramides containing nonhydroxy fatty acids and 6-hydroxysphingosines (NH) were shorter in the exacerbated AD compared to those in the maintained AD (104). Thus, SC ceramide profiling, particularly NDS, NS, and NH chain lengths, may serve as potential biomarkers for AD remission and predicting future exacerbations (104). Despite its promise, the clinical application of SC ceramide profiling remains under investigation. While liquid chromatography-mass spectrometry provides precise ceramide analysis, its use in routine practice is limited by cost, equipment requirements, and technical expertise. Future advancements in non-invasive, cost-effective analytical methods will be crucial for its integration into dermatological assessments. Combining SC ceramide profiling with other biomarkers, such as TARC, may further enhance its clinical utility, allowing for timely, objective treatment adjustments.

Stratum corneum hydration (SCH) is one of the commonly used parameters to evaluate skin barrier function (105). SCH levels in lesional skin have shown moderate predictive value for sensitization to food allergens in children with AD (106). We observed that lower increases in SCH on noninvolved skin and on eczematous lesions are factors that predict failure to dupilumab, independently of eczema area and severity index at baseline and treatment duration (107). As a non-invasive and simple method, SCH measurement of skin barrier function is safer and has shown that epidermal changes could be happening before clinical changes (108), making it a valuable predictor of treatment outcomes.

## 6 NK and NKG2D

Reduced frequency and impaired cytolytic function of circulating natural killer (NK) cells contribute to AD pathogenesis (109). Murine studies suggest that NK cells mitigate eczema and allergic inflammation by counteracting type 2 inflammation via interferon-γ (110), while NK cell deficiency exacerbates skin inflammation (109). Notably, IL-15 superagonist therapy has been shown to improve AD-like disease in mice (109), underscoring NK cell modulation as a potential immunotherapeutic strategy. AD patients exhibit NK cell deficiencies with diagnostic value that improve with treatment. A longitudinal study of children with AD found that more severe disease and increased allergen sensitivity were associated with a selective loss of NKG2D expression on NK cells, while other immune cells remained unaffected. Additionally, decreased NKG2D expression on NK cells was associated with impaired cytolytic function but an increased release of the proinflammatory cytokine tumor necrosis factor-α. Furthermore, lower NKG2D expression correlated with compromised skin barrier integrity, as indicated by increased transepidermal water loss. Reduced NKG2D expression also impaired the ability of NK cells to combat infections. Moreover, NKG2D expression on NK cells was inversely associated with AD severity and the presence of allergic comorbidities. NK cell dysregulation in AD may play a role in the progression of the atopic march (111). Low NKG2D expression provides novel cytological insights into AD initiation and progression, which may have adverse effects on prognosis.

An in-depth analysis of biomarkers in relation to the established factors contributing to poor prognosis in AD is essential. Biomarkers such as TARC, CCL18, and LDH levels are closely linked to disease severity, relapse rates, and treatment efficacy. Elevated TARC and CCL18 levels have been associated with persistent inflammation and inadequate treatment response, while high LDH and IgE levels predict poorer long-term outcomes. These biomarkers reflect the influence of environmental triggers, infections, and impaired skin barrier, which exacerbate AD. Understanding the interplay between these factors and biomarkers can guide personalized treatment strategies for AD, improving patient prognosis.

## 7 Discussion

In summary, mutations in FLG, SPINK5, STAT, KIF3A, claudin-1, Ovol1, and HLA-DRB1 genes contribute to AD susceptibility, persistence, and severity. These genetic factors affect skin barrier function, immune responses, and disease progression, highlighting the complexity of AD’s genetic basis. AD involves various endotypes with distinct inflammatory pathways, influenced by disease stage, age, and ethnicity. Obesity, environmental factors, infections, and psychological stress exacerbate the condition. Adherence enhancement, patient education, and the management of comorbidities like infections are key to improving AD outcomes. Biomarkers not only assist in assessing the severity of AD but also predict the long-term prognosis of patients, offering more precise individualized treatment strategies. A comprehensive assessment combining multiple biomarkers can help clinicians manage AD more effectively, improve treatment outcomes, and reduce the risk of relapse.

## Glossary

AD: Atopic dermatitis
EDN: Eosinophil-derived neurotoxin
FLG: Filaggrin gene
HLA: Human leukocyte antigen
HND: Head and neck dermatitis
IgE: Immunoglobin E
IL: Interleukin
IL-18R: Interleukin-18 receptor
KIF3A: Kinesin family number 3A
METRNL: Meteorin-like protein
NDS: Ceramides containing nonhydroxy fatty acids and dihydrosphingosines
NH: Ceramides containing nonhydroxy fatty acids and 6-hydroxysphingosines
NK: Natural killer cells
NS: Ceramides containing nonhydroxy fatty acids and sphingosines
SC: Stratum corneum
SCH: Stratum corneum hydration
STAT: Signal transducer and activator of transcription
TARC: Thymus and activation-regulated chemokine
TCS: Topical corticosteroids
Th: T helper cell type
TJs: Tight junctions
TSW: Topical steroid withdrawal syndrome
AhR: Aryl hydrocarbon receptor
CCL: Chemokine C-C motif ligand
DCs: Dendritic cells
EASI: Eczema area and severity index
LDH: Lactate dehydrogenase
MDC: Macrophage-derived chemokine
PARC: Pulmonary and activation-regulated chemokine
VEGF: Vascular endothelial growth factor
